# Regular, in-situ, team-based training in trauma resuscitation with video debriefing enhances confidence and clinical efficiency

**DOI:** 10.1186/s12909-018-1243-x

**Published:** 2018-06-07

**Authors:** Alexander Knobel, Daniel Overheu, Matthias Gruessing, Ingke Juergensen, Johannes Struewer

**Affiliations:** 1 Department of Orthopaedics and Trauma Surgery, Carl von Ossietzky Universität Oldenburg, Klinikum Oldenburg, Rahel-Straus Str. 10, 26133 Oldenburg, Germany; 2 Department of Anaesthesiology, Carl von Ossietzky Universität Oldenburg, Klinikum Oldenburg, Rahel-Straus Str. 10, 26133 Oldenburg, Germany

**Keywords:** Advanced trauma life support, ATLS, Simulation training, Video-debriefing, In situ simulation, Trauma resuscitation

## Abstract

**Background:**

To assess the clinical impact of a regular, multidisciplinary, video debriefed training intervention for trauma team members on real trauma resuscitations. In addition, attending personnel evaluated the training program via questionnaire.

**Methods:**

The training intervention is a regular (monthly), video debriefed, team-based trauma simulation. Training takes place in the fully functional resuscitation bay (in-situ) of the Department of Traumatology at the Klinikum Oldenburg (Level 1, primary teaching hospital for the Carl von Ossietzky University Oldenburg) involving a complete trauma team. Laerdal® Resusci Anne® dummy serves as the patient simulator. A special feature is a structured video debriefing of each participating team to analyse team performance. Data before and after implementation of training was retrospectively analysed.

**Results:**

We found a significant decrease in the time from arrival of the patient to computer tomography (CT, Spearman rank coefficient *r* = − 0.236, *p* = 0.001). Evaluation of the questionnaire by team members described a significant increase in self-confidence (*p* < 0.05).

**Conclusion:**

Monthly video assisted team based in situ training with video debriefing significantly reduces resuscitation time in the emergency bay.

## Background

Optimal care of trauma victims includes a team-based, systematic approach to assessment and therapy [1]. The interdisciplinary education of the team-members poses a challenge for trauma centres; constant training and re-evaluation of skills and process quality are an integrative component of overall trauma care. There are several guidelines to provide good quality of trauma resuscitation and education such as the “Whitebook” (s3-guideline) by the German Society for Trauma Surgery (DGU) or “Resources for the Optimal Care of the Injured Patient” by the American College of Surgeons Committee on Trauma [1–3]. Advanced Trauma Life Support (ATLS®) is a worldwide acknowledged algorithm standardizing the resuscitation of severely injured patients. The term ´trauma resuscitation´ describes the process of early systematic evaluation and treatment of life threatening conditions using a primary and secondary survey [4, 5].

Trauma resuscitation is a multidisciplinary and multiprofessional every day process within each trauma unit. Regular simulation based training - increasing technical and non-technical skills - remains mandatory to improve the algorithm compliance of each team member. Short trauma resuscitation times lead to a better chance of survival [6]. Clear communication and coordination within the trauma resuscitation team plays an important – although often neglected – role [7, 8]. Training should focus on developing the skills and the consistent presence of a trauma leader, as this seems to improve performance of trauma resuscitation [9–11].

Simulation-based training interventions offer a positively evaluated possibility to enhance skills in recognizing and dealing with emergencies [12, 13]. The term in-situ implies that a simulation training takes place in the emergency bay. In-situ simulation programs for trauma resuscitation have been evaluated positively [8, 14]. Meanwhile, the use of video assisted training programs is implemented in trauma care. Other study groups could show that debriefing is an important tool to increase simulation based learning, while video assisted debriefing is superior to verbal feedback [15–17]. However, most training programs provided by various societies educate only single team members who are often separated from each other. In-house training programs are therefore increasingly implemented.

In order to combine all potential advantages of training components, we found it necessary to create a training intervention with a unique combination of different elements. We established a monthly training program within our real trauma resuscitation bay (in-situ) involving a complete trauma team. As an additional feature, the training sessions are video recorded and a video assisted debriefing is part of each session. One objective of the training is an efficient and structured transfer of the patient from pre-hospital-environment to the emergency department without loss of information. Technical skills, e.g. airway management, application of chest tube or IV line, Focused Assessment with Sonography in Trauma (FAST) [18], are also included.

Primary objective of the present study is therefore: Is there a positive impact of team-based real time video assisted in situ training on the quality of real patient care? Another component of trauma resuscitation and quality management is the confidence of team members. To gain feedback from the participating staff about the monthly video assisted training sessions, a one-time questionnaire supplemented the training program.

## Methods

### Design

This is a retrospective analysis of the effect of a regular multidisciplinary in situ trauma simulation training intervention in a Level 1 Trauma Centre in Germany. Data of real trauma resuscitations before and after the implementation of the training was assessed. The basic team for trauma resuscitation is assembled by colleagues of several departments: Traumatology (17 colleagues), Anaesthesiology (60 colleagues), Emergency Nurses / Intensive Care Nurses (each 40 colleagues) and Radiology (10 colleagues). Together there are approximately 190 colleagues taking part in trauma resuscitation.

The training was established in July of 2013. We defined a time period of pre-training of one year and a post-training period of two years. The monthly training intervention took place 24-times within this period. The exposure of colleagues to the training program varied by department between 100% (Traumatology, Emergency Nurses and Radiology) and approximately 40% (Anaesthesiology). The single team members for each training-session were scheduled by the participating departments.

### Training intervention

The evaluated training represents an in-situ *training* under real time conditions taking place in the real resuscitation bay of the emergency department. As in the case of real trauma resuscitation, all devices and materials are facilitated (e.g. airway management materials, IV lines, chest tubes, wound care materials, splints, pelvic binders). These monthly scenarios are selected and worked out by two tutors (physician and nursing staff). According to the DGU Whitebook, the scenario team consists of two trauma surgeons (at least one consultant), one anaesthetist (consultant), one radiologist, and three nursing staff members [2].

A real time resuscitation is performed. The role of the trauma leader is assigned to the consultant of trauma surgery and signalled by wearing a special labelled vest. Depending on the scenario further team members of other specialties (e.g. general surgeon, paediatrician, heart surgeon each plus nursing staff) are invited by the tutor based on the fundamentals of trauma care [1]. Furthermore, to simulate patient and information hand-over, paramedics are integrated into the scenario.

Video recording equipment is set up before each training session. The patient is simulated by a training dummy (Laerdal® Resusci Anne®, Sim Pad®, LLEAP® (Laerdal Learning Application)). Features are breathing sounds, electrocardiogram (ECG) and possible defibrillation, airway management, blood pressure and cardiopulmonary resuscitation (CPR) feedback. An additional tutor controls the computer screen displaying further patient related information, such as vital signs, ultra sound pictures etc.

The team members gather in the resuscitation bay. Similar to real life emergencies, the nursing staff initially receives a simulated phone call announcing the patient and giving a rough estimate of the trauma mechanism and anticipated injuries. After five minutes of preparation time the patient simulator is admitted by paramedics and accompanied by an emergency physician. Now the performing team has a fixed fifteen-minute period for patient hand over and to conduct trauma resuscitation. The training cut off is set when either the patient is stabilized and ready for transport to CT scan / necessity of emergency operation or when the given time is over.

All team members and additional staff of trauma surgery, anaesthesiology, radiology and nursing staff attend a debriefing after each training session. The video recording of each stage of the resuscitation is replayed separately.

- Preparation
- Hand over
- Initial assessment (primary survey)
- Secondary assessment (secondary survey)
- Additional procedures

During the review, emphasis is put on situational awareness, compliance to the ATLS algorithm, decision-making, teamwork and communication. Special attention is given to the role of the trauma leader.

### Clinical data

The time from admission of the patient to CT equals resuscitation time. Although it is not a direct parameter for quality of patient care, it is a crucial clinical parameter for efficiency in the emergency bay [1, 2, 19].

Data was extracted from the documentation for the German trauma registry of our department. The following additional parameters were gathered to describe the patient population pre- and post-training:

- Age
- Sex
- Date of admission
- Mechanism of injury
- Injury Severity Score (ISS)

A correlation analysis (Spearman rank correlation) of the CT transfer time for the whole 3-year period (pre- and post-training) was performed.

### Training staff survey

*Training staff survey*: We investigated the effect of our monthly video assisted in situ training on our staff. This was achieved by a one-time survey referring to the training program which was answered by training participants of all involved specialties. A five-part Likert scale was used ranging from ´one´ for complete approval to ´five´ for complete disapproval.

- Q1) “The objective of the training intervention is stated clearly.”
- Q2) “The scenario is realistic.”
- Q3) “Debriefing and discussion of the scenario was helpful.”
- Q4) “The debriefing was professional; no one was offended.”
- Q5) “The debriefing gave me the possibility to recognize my mistakes.”
- Q6) “I am able to fulfil my responsibilities during trauma resuscitation.”
  - Q6.1) pre-training
  - Q6.2) post-training
- Q7) “I am sufficiently prepared for trauma resuscitation.”
  - Q7.1) pre-training
  - Q7.2) post-training
- Q8) “I will apply my newly gained skills in the future.”
- Q9) “I would recommend that all members of the involved departments participate in the training.”

All colleagues of the participating departments received a survey (in-house email): 71 questionnaires were returned (return rate ≈ 37%), 8 surveys were not filled in correctly (e.g. double marks within the Likert scale) and 63 questionnaires (88.7% of 71) could be evaluated (Anaesthesiology (21), Trauma surgery (15), Radiology (4), Nursing staff (23).

### Statistics

For statistical analyses IBM® SPSS® 23 for Windows® was used. The level of significance for all tests was set to *p* ≤ 0.05. All tests for significance were two-sided, a confidence interval of 95% was used for all calculations. Times of resuscitation were evaluated using a Spearman rank correlation coefficient (r_s_) with a two-sided p-test. The training staff survey was evaluated by descriptive statistics. The ´before´ and ´after´ questions 6 and 7 were each assessed by a Wilcoxon signed-rank test to compare two matched samples. Patient population pre- and post-training was assessed with Mann-Whitney-U test and Fisher’s exact test. Mean values are stated including standard deviation (SD).

## Results

We were able to identify 184 cases of trauma resuscitation between July 2012 and June 2015. The pre-training subgroup consisted of 48 patients versus 136 cases post-training. Injury Severity Score (ISS) was calculated for each patient [20]. Table 1 compares the characteristics of the patient population pre- and post-training. There are no significant differences.

**Table 1:** Characteristics of the patient population pre- and post-training. Mann-Whitney-U test for ISS, age; Fisher’s exact test for age, blunt trauma and craniocerebral trauma

		pre-training	post-training	*p*-value
ISS	mean:	25.6	26.1	0.84
	SD:	11.6	13.3	
	Median:	24	24.5	
age	mean:	38.6 years	39.4 years	0.78
	SD:	20.6	21.4	
sex	male:	72.9% *n* = 35	70.6% *n* = 96	0.85
	female:	27.1% *n* = 13	29.4% *n* = 40	
blunt trauma		95.8% *n* = 46	94.9% *n* = 129	1
craniocerebral trauma		16.7% *n* = 8	16.9% *n* = 23	1
n		48	136	

### Resuscitation time / transfer time to CT diagnostics

**Fig. 1:**
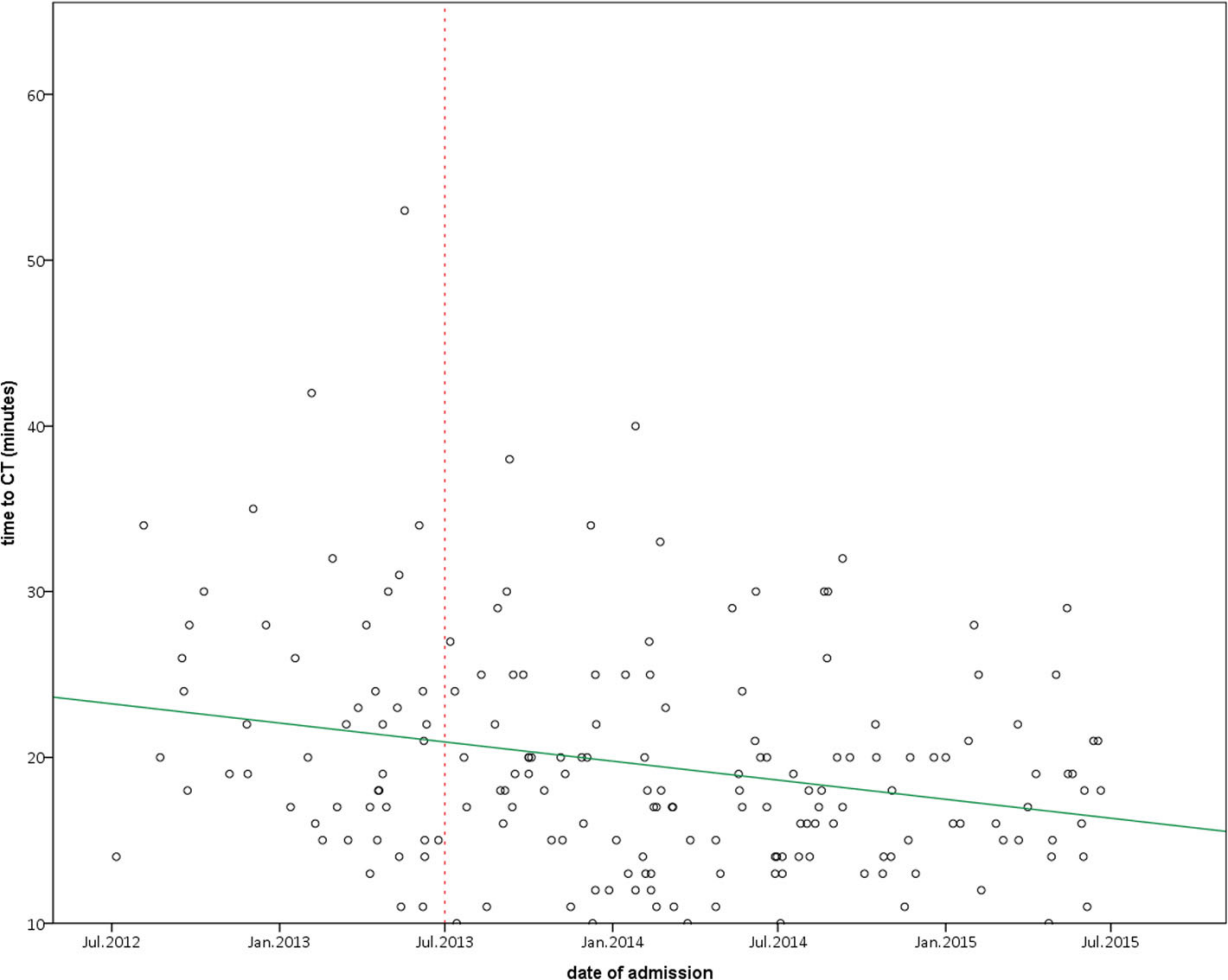
Time to CT in correlation to date of admission; green line: balance line to visualize decrease; red line: marks date of implementation of training intervention

We found a significant decrease in the time from arrival of the patient to the start of CT imaging. The median for the 48 cases before training started was 22.5 min (mean 22.3 min, SD 8.3), whereas this time decreased to 18 min (mean 18.6 min SD 6) for the 136 cases after July 2013. The correlation analysis showed a significant decrease (Spearman rank correlation between *date of admission* and *time to CT*, r_s_ = − 0.236, *p* = 0.001, Fig. 1).

### Training staff survey

*Training staff survey*: Staff survey questions Q1 to Q9 are listed under Methods. The values for median of all items of the questionnaire are given in Table 2. All given answers within each item of the questionnaire ranged from 1 to 5.

**Table 2:** Summarized results of staff survey, questions 6.1/6.2 and 7.1/7.2 were assessed by Wilcoxon signed-rank test

summary staff survey											
	question										
	Q1	Q2	Q3	Q4	Q5	Q6.1	Q6.2	Q7.1	Q7.2	Q8	Q9
median	2	1	1	1	1	3	1	3	1	1	1
*n* = 63						*p* < 0.05		*p* < 0.05			

Questions 6 and 7 target subjective knowledge and self-confidence before and after training. Each question was tested pre-training versus post-training using Wilcoxon signed-rank (for both *p* < 0.05, Table 2*).* A significant increase for both parameters could be shown.

## Discussion

The main purpose of the present study was to evaluate the clinical impact of a monthly video debriefed in-situ trauma simulation. We could demonstrate a significant decrease of trauma resuscitation time after the training was implemented.

Participating staff assessed the monthly training as a realistic and productive method; in particular, the video debriefing was rated very positively. They recommended that further staff should be trained this way. Self-assessment showed a significant increase of self-confidence in trauma resuscitation and knowledge.

### Limitations

Did our training intervention cause the downward trend in resuscitation time?

There can be no statistical proof for a relation between decrease in resuscitation time and the training intervention.

However, our training intervention has been the main multidisciplinary measure to improve the quality of patient care between July 2012 and June 2015 in our hospital. Hospital staff have attended the required courses (e.g. ATLS®-courses) for a trauma centre for years before and after implementation of our training intervention [1, 2]. We do not consider the single staff member, but a multidisciplinary group. Each team is assembled ad hoc with consistently rotating participants - that is the nature of trauma resuscitation. Constant monthly training spreads the knowledge and non technical skills throughout the workforce. More than 160 colleagues (out of 190) have had the opportunity to participate in our training. Confounding factors like employee turnover were present before and after the training was implemented – therefore they may be ignored. This study is a retrospective evaluation of trauma records. The launch of the training program was not accompanied by additional monitoring or documentation for real trauma resuscitations. The Hawthorne effect can be neglected.

The decrease in resuscitation time is statically significant, but is it clinically significant?

Speed is not the most important metric in trauma resuscitation, but it is a useful parameter for efficient trauma care [1, 19]. Other studies implicate that there are other useful parameters which could be assessed to show the effectiveness of trauma resuscitation (Mortality, time to intubation, time to chest tube, etc.). Our study’s retrospective design allowed only a limited evaluation of the processes in our emergency department. Most of these parameters were simply not recorded permanently enough in our daily routine in the emergency department and therefore are lost to possible evaluation.

### Interpretation

The distinctiveness of this study is the combination of constant in situ trauma resuscitation simulation training with a video debriefing. The effectiveness of video registration and simulation training for trauma resuscitations has been indicated by many authors [8, 21, 22]. Scherer et al. showed that videotaped performance reviews of real trauma resuscitations increased the learning effect compared to verbal feedback only [17]. They videotaped and reviewed real trauma resuscitations throughout a six-month period. For three months only, verbal feedback was given. Within the remaining three months, teams attended a video debriefing. The video debriefing group showed significant and lasting changes in trauma performance and behaviour. It was therefore implicated that videotape reviews improve algorithm compliance and patient care. They strongly support the video debriefing component of our study, which we found extremely useful to evaluate the team performance.

Steinemann et al. evaluated the effect of a single (four-hour) multidisciplinary in situ training simulation with video debriefing [23]. Real trauma resuscitations were assessed six months before (*n* = 141) and six months after (*n* = 103) the training intervention. They showed that even this relatively brief intervention had a positive effect on team performance. Similar to our study, the trauma resuscitation time decreased significantly. However, compared to our study, only a single training intervention was conducted, whereas we performed a continuous monthly training over a period of two years.

## Conclusion

The rapid completion of trauma resuscitation is paramount to reduce morbidity and mortality. It is a multidisciplinary challenge for every hospital to train and educate the team members. Regular in situ training with video debriefing is an important tool to maintain a good and fast trauma resuscitation algorithm, and has been implemented in our emergency bay since July of 2013. We will continue our monthly training program. Other quality oriented parameters of patient care in the emergency bay need to be thoroughly recorded. Possible future studies will involve video assisted pre-hospital training.

## Abbreviations

ATLS®: Advanced Trauma Life Support
CPR: Cardiopulmonary resuscitation
CT: Computer tomography
DGU: German Society for Trauma Surgery (Deutsche Gesellschaft für Unfallchirurgie)
ECG: Electrocardiogram
FAST: Focused Assessment with Sonography in Trauma
ISS: Injury Severity Score
LLEAP®: Laerdal Learning Application
